# Characterisation of the epidemic strain of H3N8 equine influenza virus responsible for outbreaks in South America in 2012

**DOI:** 10.1186/s12985-016-0503-9

**Published:** 2016-03-19

**Authors:** Edsel Alves Beuttemmüller, Alana Woodward, Adam Rash, Luis Eduardo dos Santos Ferraz, Alice Fernandes Alfieri, Amauri Alcindo Alfieri, Debra Elton

**Affiliations:** Universidade Estadual de Londrina, Rodovia Celso Garcia Cid - Pr 445 Km 380, Campus Universitário, Londrina, Paraná CEP 86057-970 Brazil; Animal Health Trust, Lanwades Park, Kentford, Suffolk CB8 7UU UK; Laboratórios Vencofarma do Brasil, Travessa Dalva de Oliveira, 237, Londrina, Paraná CEP 86030-370 Brazil

**Keywords:** Equine influenza, Epidemic, Haemagglutinin, Neuraminidase, Florida clade 1, Brazil

## Abstract

**Background:**

An extensive outbreak of equine influenza occurred across multiple countries in South America during 2012. The epidemic was first reported in Chile then spread to Brazil, Uruguay and Argentina, where both vaccinated and unvaccinated animals were affected. In Brazil, infections were widespread within 3months of the first reported cases. Affected horses included animals vaccinated with outdated vaccine antigens, but also with the OIE-recommended Florida clade 1 strain South Africa/4/03.

**Methods:**

Equine influenza virus strains from infected horses were isolated in eggs, then a representative strain was subjected to full genome sequencing using segment-specific primers with M13 tags. Phylogenetic analyses of nucleotide sequences were completed using PhyML. Amino acid sequences of haemagglutinin and neuraminidase were compared against those of vaccine strains and recent isolates from America and Uruguay, substitutions were mapped onto 3D protein structures using PyMol. Antigenic analyses were completed by haemagglutination-inhibition assay using post-infection ferret sera.

**Results:**

Nucleotide sequences of the haemaglutinin (HA) and neuraminidase (NA) genes of Brazilian isolate A/equine/Rio Grande do Sul/2012 were very similar to those of viruses belonging to Florida clade 1 and clustered with contemporary isolates from the USA. Comparison of their amino acid sequences against the OIE-recommended Florida clade 1 vaccine strain A/equine/South Africa/4/03 revealed five amino acid substitutions in HA and seven in NA. Changes in HA included one within antigenic site A and one within the 220-loop of the sialic acid receptor binding site. However, antigenic analysis by haemagglutination inhibition (HI) assay with ferret antisera raised against representatives of European, Kentucky and Florida sublineages failed to indicate any obvious differences in antigenicity.

**Conclusions:**

An extensive outbreak of equine influenza in South America during 2012 was caused by a virus belonging to Florida clade 1, closely related to strains circulating in the USA in 2011. Despite reports of vaccine breakdown with products containing the recommended strain South Africa/03, no evidence was found of significant antigenic drift. Other factors may have contributed to the rapid spread of this virus, including poor control of horse movement.

**Electronic supplementary material:**

The online version of this article (doi:10.1186/s12985-016-0503-9) contains supplementary material, which is available to authorized users.

## Background

Equine influenza is one of the major infectious respiratory diseases in equids worldwide. The causative agent, equine influenza virus (EIV), is a highly contagious pathogen endemic in most parts of the world. Like other influenza A viruses, EIV has two surface glycoproteins, haemagglutinin (HA) and neuraminidase (NA). HA plays an essential role in virus entry by attaching to host cell sialic acid receptors and promoting membrane fusion [1]; it is a major target of neutralizing antibodies and is therefore an important component of commercial vaccines. NA has sialidase activity and is thought to play a role in virus entry as well as exit. Antibodies elicited against influenza NA are also known to contribute to protection and have recently been shown to block both sialidase activity and virus adsorption [2], however their importance for immunity against EIV remains unclear.

Two influenza subtypes are known to infect horses, H7N7 and H3N8. H7N7 equine influenza was first isolated in Europe in 1956, followed by H3N8 in 1963 [3, 4]. The two subtypes co-circulated, with reassortment, until the last isolation of H7N7 in the late 1970s [5, 6]. Following its emergence in South America, most likely from an avian source EIV H3N8 diverged phylogenetically into American and Eurasian lineages [7, 8], with sufficient antigenic differences to warrant inclusion of both in vaccines. Subsequently three sub-lineages named Kentucky, Florida and South American (Argentinian) emerged within the American lineage [9], and the Florida sublineage has further diverged into distinct clades 1 and 2 [10, 11]. Currently, viruses from the Florida sub-lineage are prevalent worldwide and inclusion of representatives of both clade 1 and clade 2 are recommended for vaccines [12]. Florida clade 1 (FC1) viruses are considered endemic in North America, but have caused major outbreaks in Australia, Japan and South Africa [13–15]. There have also been smaller outbreaks reported in Europe from 2007 to 2009 [16–18]. In Asia and Europe viruses from Florida clade 2 (FC2) predominate, causing significant outbreaks in China, India and Mongolia in recent years and smaller scale outbreaks in multiple countries of Europe [19–23].

Vaccination has been widely used for the control of equine influenza and commercial vaccines have been available for several decades. Despite this, horses immunized with potent vaccines occasionally fail to provide adequate protection. Vaccine breakdown can occur as a consequence of antigenic drift in HA [24, 25], a well-established phenomenon amongst mammalian influenza A viruses. Cumulative mutations over time result in amino acid substitutions in HA, allowing eventual immune escape of emerging strains from the humoral protection of the host, previously acquired against earlier strains [26, 27]. Additionally, reassortment among gene segments during mixed infections of influenza viruses can contribute to the appearance of new strains, a process known to have occurred amongst recent EIV strains from different sub-lineages [10, 11]. To counteract the effects of antigenic drift, surveillance and vaccine strain selection are carried out for EIV. In 2010 the World Organisation for Animal Health (Office International des Epizooties, OIE) updated the recommendations for suitable antigens for commercial vaccines to include a representative strain from both Florida clade 1 and Florida clade 2. However, many vaccine manufacturers still provide products with outdated vaccine strains.

During 2012, an extensive outbreak of equine influenza spread through South America, first reported in Chile in December 2011 and affecting Brazil by February 2012. The outbreak spread rapidly through Chile, Brazil, Uruguay and Argentina [28, 29] but it is thought likely that other countries in South America were also affected. Horses carrying EIV were transported from Uruguay to Dubai, highlighting the risk to other countries [23]. Prior to this outbreak, the most recent Brazilian EIV strain to undergo molecular characterisation was A/eq2/Brazil/1987 [30]. This paper describes the 2012 Brazilian outbreak and the molecular and antigenic characterisation of a representative EIV isolate, A/equine/Rio Grande do Sul/2012.

## Methods

### Collection of samples

Nasopharyngeal swabs were collected from horses showing typical influenza clinical signs, including nasal discharge, cough and pyrexia. All sampled horses were aged between 2 and 3 years old and only horses within the first 3 days of the onset of clinical signs were sampled. Clinical samples were processed for diagnostic testing at Universidade Estadual de Londrina, Brazil with viruses isolated in fertile hen’s eggs and MDCK cells. Further analyses of egg-grown virus isolates were completed at the Animal Health Trust, UK.

### EIV diagnosis: RNA detection from clinical samples

Purification of RNA from swab samples was performed using guanidinium thiocyanate and silica particles [31]. For detection of EIV RNA, a two step RT-PCR was employed using equine-specific H3 HA primers [32]. Reverse transcription (RT) was carried out using SuperScript III Reverse Transcriptase (Life Technologies) according to manufacturer’s recommendations at 55 °C. PCR amplification was performed using Platinum® Taq DNA Polymerase (Life Technologies).

### Virus isolation

Virus isolation was attempted from RT-PCR positive samples, simultaneously in embryonated hens’ eggs and Madin-Darby canine kidney cells (MDCKs). For isolation in eggs 0.1 ml of each swab sample was inoculated into the allantoic fluid of 11-day-old embryonated SPF eggs, as described previously [10]. For isolation in MDCKs, cells were cultured in 25 × 145 mm tubes and rotated. Before inoculation, samples were pre-treated with 2 μg/ml of TPCK-trypsin for 30 min at 37 °C and the cells were washed three times with PBS containing 0.5 μg/ml of TPCK-trypsin. After inoculation, the tubes were incubated for 90 min on a roller at 33 °C. The inoculum was discarded and cells washed with sterile PBS before adding serum-free Earle’s medium containing antibiotics and 0.5 μg/ml of TPCK trypsin. After 72 h, tubes were frozen at −80 °C. All samples were subjected to two passages in eggs and cells under the conditions described above.

### Genetic analyses

RNA was extracted from low passage egg-grown isolates using a QIAamp Viral RNA Mini Kit (Qiagen) according to manufacturer's instructions. Following RT with Superscript II and the UNI-12 primer [33], PCR products suitable for sequence analysis were generated using gene specific primers tagged with M13 primer sequences as described previously [34]. Amplification products were visualized on 1 % agarose gels with GelRed nucleic acid stain (Biotium) then purified using a QIAquick PCR purification kit (Qiagen) according to manufacturer’s directions. Sequencing was completed on an ABI PRISM® 3100 Genetic Analyzer (Applied Biosystem) using BigDye Terminator v3.1 (Applied Biosystem). Nucleotide sequences were edited using SeqMan II, version 5.03 (DNAstar Inc.) and BioEdit (Ibis Pharmaceuticals Inc.). Alignments were performed using ClustalW2 (EMBL-EBI) and Maximum-Likelihood trees of nucleotide sequences for each segment were constructed using PhyML version 3.

### Antigenic characterization

*S*erum was raised against A/equine/Rio Grande do Sul/2012 in two co-housed 6 month old female ferrets by intranasal instillation of 0.1 ml of a limiting dilution egg-grown stock of EIV per nostril, equivalent to a final dose of 2 × 10^6^ EID_50_. Sera were collected 3 weeks post-infection and stored at −20 °C. The remaining ferret antisera were from AHT archives. Prior to use, antisera were treated with potassium periodate, 3 % glycerol-PBS added and heat-inactivated at 56 °C for 30 min. Haemagglutination inhibition (HI) assays were carried out in V-bottomed 96-well plates, as described previously [23]. HI titres were expressed as the reciprocal of the highest dilution to show 100 % inhibition of haemagglutination.

### Ethical approval

Ferret sera were raised under a UK Home Office project licence. All work underwent full ethical review at the Animal Health Trust, by 3Rs and the Animal Welfare and Ethical Review (AWERB) committees and complied with the revised Animals (Scientific Procedures) Act 1986 under the EU Directive 2010/63/EU.

## Results

### Outbreak description

**Fig. 1:**
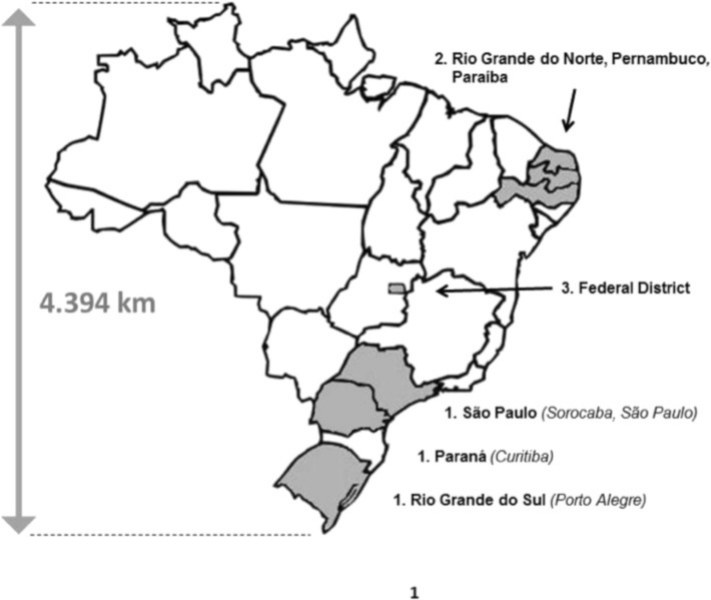
Map of Brazil showing the distance in kilometers between the extreme points of the country, and indicating the states where the outbreak was reported in chronological order: March (1), April (2) and May (3)

During 2012 equine influenza outbreaks were reported throughout South America, including Chile, Brazil, Uruguay and Argentina ([23, 28, 29]; this manuscript). Virus was first isolated from horses in the state of Rio Grande do Sul, in the extreme south of Brazil (Fig. 1). Clinical cases of EIV were first reported in February, following an International Creole Rodeo held in Vacaria. Shortly after the event finished, further cases were reported in Porto Alegre and races were cancelled due to the poor health status of the horses. On 7^th^ March, nasopharyngeal swabs were collected from six Thoroughbred horses at the Jockey Club of Porto Alegre. Within 24 h, influenza-like clinical signs were reported further north at the Jockey Club of Sorocaba, Sorocaba city, in the state of São Paulo (Fig. 1). Horses presented with pyrexia ranging from 39 to 41 °C and five Quarter Mile horses and one Thoroughbred were sampled. Samples collected from both sites were positive for EIV by RT-PCR.

In late March a barrel racing competition was held near Sorocaba city, with approximately 1,200 Quarter Mile horses participating from locations throughout Brazil. Anecdotal reports indicated that sick animals were present at the event, later press reports indicated that equine influenza had subsequently spread to the southeast, midwestern and some northeastern states of Brazil. Press reports from 10 and 26^th^ April, 2012, stated that the disease had spread to the states of Pernambuco, Paraiba and Rio Grande do Norte after a competitive vaquejada event (Fig. 1). On 15 May, 2012 in the Federal District, the press reported that an ongoing exhibition event was cancelled due to EIV infection. Around 100 rural properties had horses showing clinical signs and around 600 horses from local breeders were affected. The Jockey Clubs of Sorocaba and Rio de Janeiro were not interdicted. Sorocaba had the disease under control before the arrival of the authorities and the Jockey Club of Rio de Janeiro had prohibited the entry of other animals from other jockey clubs, thus preventing the spread of EIV into its facilities.

### Diagnosis and virus isolation

EIV RNA was detected by RT-PCR directly from 8 of 12 nasopharyngeal swabs collected from two jockey clubs in two different states. Four samples were positive from Sorocaba and four from Porto Alegre. After two serial passages, virus was successfully isolated from five samples in embryonated eggs and three in MDCK cell culture. The isolates originated from one horse with unknown vaccination history, two horses vaccinated with a vaccine containing the American strain A/equine/Kentucky/97 and two horses vaccinated with a vaccine containing the Florida clade 1 strain A/equine/South Africa/4/03. The antigen and adjuvant content of the vaccines cited here are shown in Table 1. Initial isolation on MDCK cells did not result in obvious cytopathic effect (CPE), however after two further passages the virus isolates caused marked cellular destruction (data not shown).

**Table 1:** Vaccine adjuvants and virus antigens of products used to vaccinate horses sampled in this study

H3N8 antigen	H7N7 antigen	Adjuvant
Kentucky/97		Lipid-based
Kentucky/94 & South Africa/4/03	Prague/56	Aluminium hydroxide
Newmarket/2/93 & South Africa/4/03	Prague/56	Aluminium hydroxide

### Vaccination history

Four out of the six sampled horses from Sorocaba had been vaccinated with inactivated South Africa/4/03 antigen and a fifth horse with inactivated Kentucky/97. One horse had an unknown vaccination history. In Porto Alegre, four horses had unknown vaccine history and two horses had received a booster containing Kentucky/97. Of the vaccinated horses, only two had received a booster vaccination during the previous 30 days; one had a dose of South Africa/4/03- and the other a dose of Kentucky/97-containing vaccine. Both horses had pyrexia at the time of sampling (39 and 40 °C respectively). All the remaining horses with known vaccination history had received their last vaccination booster more than 4 months previously. All the swabbed horses had been vaccinated, including those where full details were not available.

### Phylogenetic analysis of genes encoding surface glycoproteins HA and NA

**Fig. 2:**
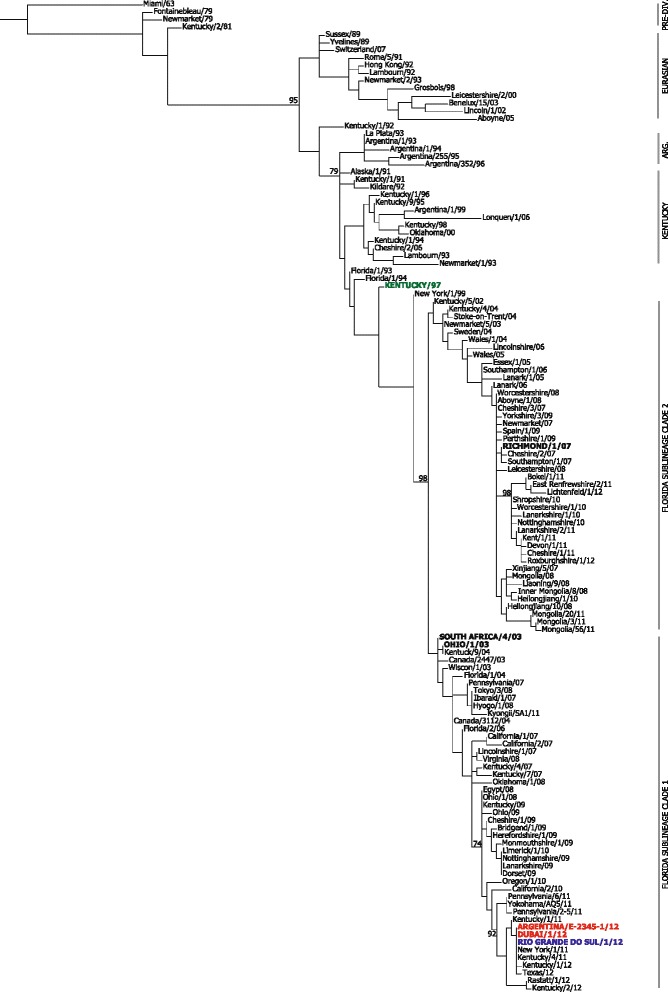
Phylogenetic analysis of HA1 nucleotide sequences from South America, 2012. Maximum likelihood tree based on EIV strains isolated between 1963 and 2012. Bootstrap values obtained after 100 replicates are shown at major nodes. Major lineages and sub-lineages are labeled by *bars* on the right. The OIE recommended strains South Africa/4/03, Ohio/03 (Florida clade 1) and Richmond/1/07 (Florida clade 2) are in *black uppercase letters*. The vaccine strain Kentucky/97 is shown in *green*, the Brazilian strain Rio Grande do Sul/1/12 in *blue*, Dubai/1/12 and Argentina/E-2345-1/12 in *red*

**Fig. 3:**
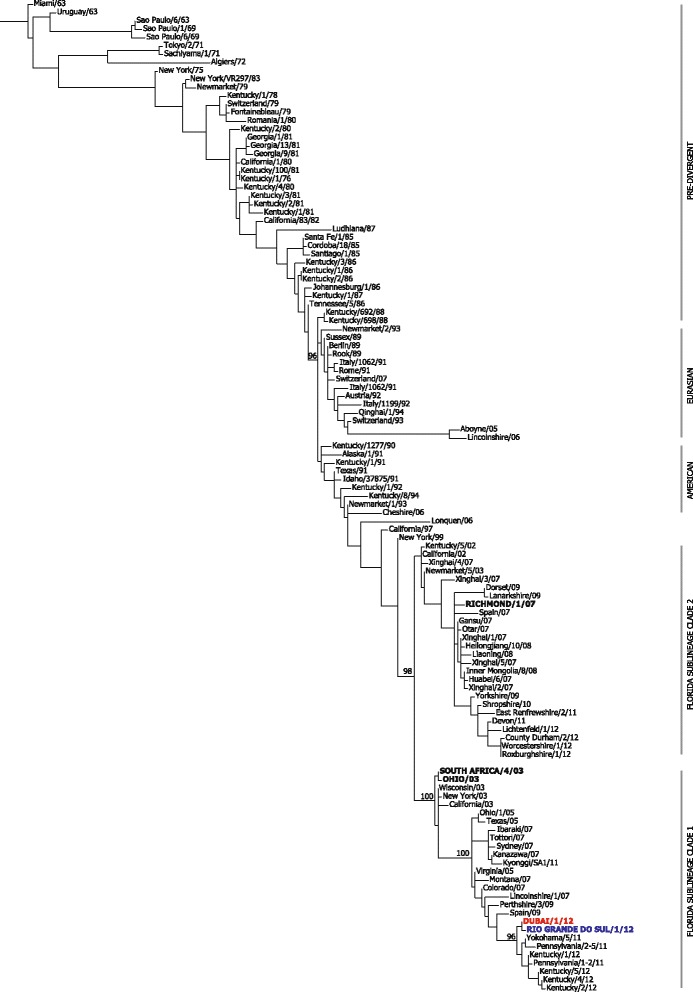
Phylogenetic analysis of NA nucleotide sequences from EIV. Maximum likelihood tree based on EIV strains isolated between 1963 and 2012. Bootstrap values obtained after 100 replicates are shown at major nodes. Major lineages and sub-lineages are shown by *bars on the right*. The OIE recommended strains South Africa/4/03, Ohio/03 (Florida clade 1) and Richmond/1/07 (Florida clade 2) are in *black uppercase letters*. Representative of the South American outbreak, Rio Grande do Sul/1/12 is shown in blue and Dubai/1/12 in *red*

**Fig. 4:**
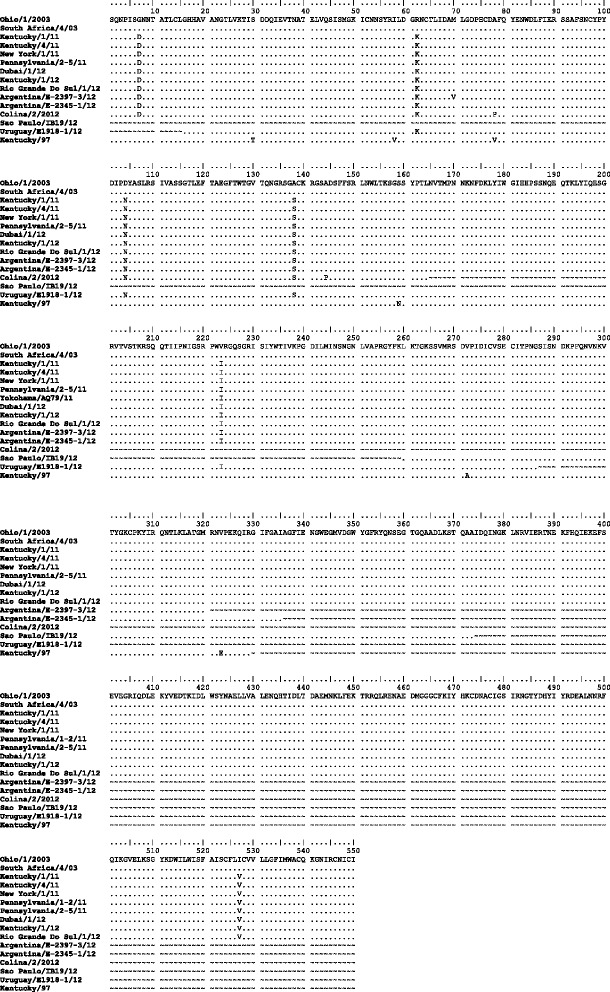
Comparison of HA amino acid sequences. Rio Grande do Sul/1/12 and 2011–12 Florida clade 1 strains from the USA were aligned to the OIE-recommended vaccine strains Ohio/03 and South Africa/4/03 strains (top). Additional complete and partial sequences available from the South American outbreak were also included for Dubai/1-3/12, Argentina/E-2397-3/12, Argentina/E-2345-1/12, Colina/2/12, São Paulo/IB19/12 and Uruguay/E-1918-1/12. Kentucky/97, a vaccine strain used during the outbreak, is also shown. Amino acid identity is represented with a *dot*

Virus was successfully isolated from two horses at Rio Grande do Sul. The nucleotide sequences covering the coding regions and the majority of the untranslated 5’ and 3’ ends were determined for all eight segments for the isolate A/equine/Rio Grande do Sul/1/12 and submitted to GISAID [35]. For comparative purposes, the whole genome was also determined for a recent clinical isolate from the USA, A/equine/Kentucky/1/11. For phylogenetic analysis, representative equine H3N8 HA and NA nucleotide sequences from the Pre-divergence, Eurasian and Kentucky lineages and sub-lineages, as well as contemporary isolates of Florida clades 1 and 2 were retrieved from GenBank and GISAID. The analysis was completed for HA1 only, due to a lack of availability of full-length sequences for HA. Maximum Likelihood Phylogenetic trees for HA1 and NA are shown in Figs. 3 & 4 respectively. Analysis of the nucleotide sequence of HA1 showed six distinct clusters corresponding to the Pre-divergent and Eurasian lineages, as well as the Argentinian, Kentucky and Florida (clades 1 and 2) sub-lineages (Fig. 2). All the 2012 sequences from South America were located within the Florida clade 1 group, including isolates from Uruguay via Dubai [23], Argentina [29] and those from Brazil reported here. They were most closely related to viruses isolated in the USA during 2011–2012 (Fig. 2). The vaccine strain Kentucky/97 was more closely related to very early Florida viruses, prior to the subdivision into clades 1 and 2, whereas South Africa/03 grouped with early representatives of Florida clade 1, as expected (Fig. 2).

The phylogenetic tree for the complete NA sequences also formed well defined clusters corresponding to equivalent lineages and sub-lineages as those described for HA1 (Fig. 3). The exception was the absence of a clear subdivision for the Argentinian sub-lineage, due to a lack of available sequence data for representative strains. Again, the isolates from the Brazilian outbreak clustered with viruses belonging to the Florida clade 1 group, in particular the USA isolates from 2011 to 2012. Unfortunately, data was not available for the vaccine strain Kentucky/97 nor the recent strain from Argentina (Argentina/E-2345-1/12).

### Comparison of HA amino acid sequences

**Fig. 5:**
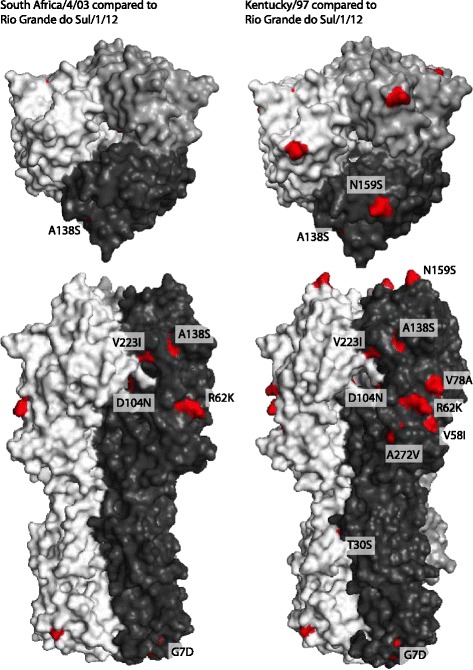
HA structure, showing location of amino acid substitutions between Rio Grande do Sul/1/12 and vaccine strains. The amino acid sequence of the outbreak strain was compared against two vaccine strains, A/equine/South Africa/4/03 and A/equine/Kentucky/97. The locations of the substitutions are indicated in *red* on the influenza H3 structure for A/equine/Richmond/1/07 (protein database number PDB: 4UO0, [44]). Structures were examined using MacPyMOL and images collected for *top* and *vertical views*

The derived amino acid sequences of HA for Rio Grande do Sul/1/12 and contemporary isolates from USA, Uruguay, Argentina and Dubai [23, 29] were compared to the Florida clade 1 OIE-recommended strains Ohio/03 and South Africa/4/03, shown in Fig. 4. Within HA1, five consistent amino acid substitutions (G7D, R62K, D104N, A138S and V223I) were present in all the recent Florida clade 1 isolates, including the sequences from South America. Of these, A138S occurred within antigenic site A and V223I occurred within the 220 loop of the receptor-binding site. Argentina/E-2397-3/12 had the additional substitution M70V however, in the absence of further sequences it is unclear whether this was common to all viruses circulating in Argentina during 2012. Two further partial HA1 sequences from Brazil (São Paulo/IB19/12, residues 260–373) and Chile (Colina/2/12, residues 1–164), were retrieved from Genbank and included in the alignment. The sequence from São Paulo was identical to the other South American strains, whereas the Chilean sequence contained two unique substitutions, A78P and A144P. Both substitutions occur within antigenic regions of H3, within sites E and A respectively. Compared with the vaccine strain Kentucky/97, Rio Grande do Sul/1/12 had 11 changes within the HA1 region and one change in HA2. The amino acid substitutions A138S, N159B and V78A were located in antigenic sites A, B and E respectively, whereas the change A272V occurred in the loop region close to antibody-binding site C. Within the region encoding HA2, a consistent substitution I527V was observed in the Rio Grande do Sul/12 and Dubai/1/12 strains compared to South Africa/4/03, also seen in the American sequences from 2011 and Kentucky/1/12. The HA2 region was not covered by the Argentinian sequences. The locations of individual mutations were mapped on the three dimensional equine H3 HA structure, shown in Fig. 5, which shows comparison of the outbreak strain against both the OIE-recommended vaccine strain Ohio/03 and Kentucky/97, both of which were in use during the outbreak.

### Comparison of NA amino acid sequences

**Fig. 6:**
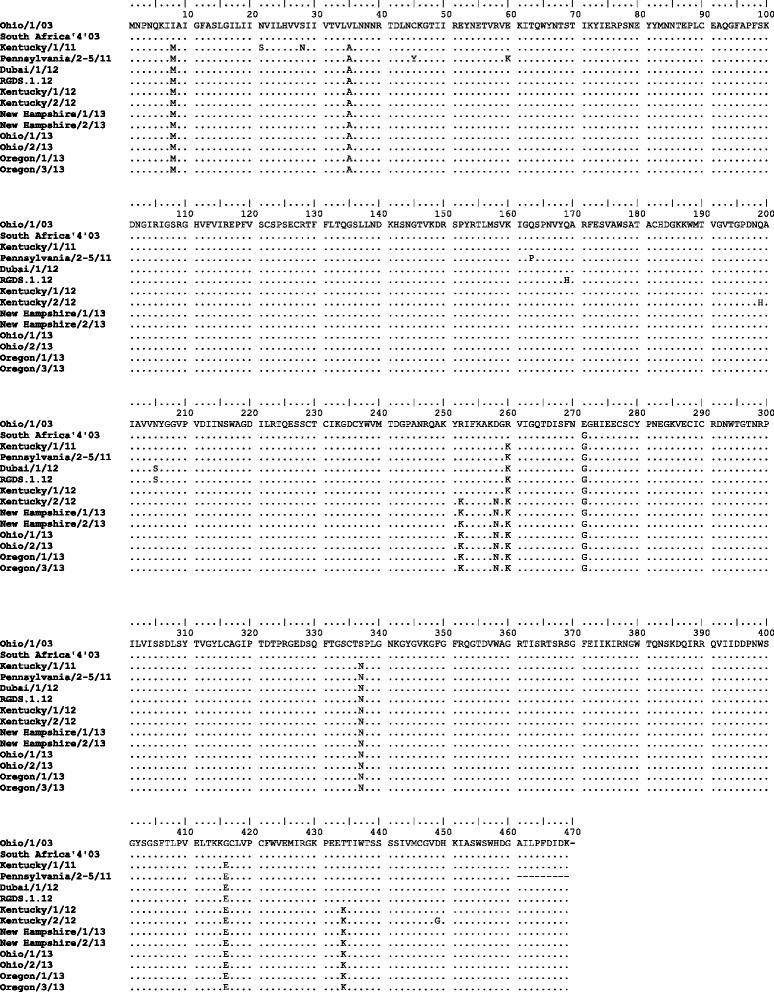
NA amino acid alignment. Amino acid sequences for Rio Grande do Sul/1/12, and 2011–13 Florida clade 1 strains from the USA were compared to the recommended OIE strain Ohio/03 (*top*). Amino acid identity is represented with a *dot*

The NA amino acid sequences of the isolate Rio Grande do Sul/1/12, Kentucky/97 and Florida clade 1 isolates from 2011 to 2012 were aligned to the OIE recommended strains Ohio/03 and South Africa/4/03, shown in Fig. 6. More recent sequences from 2013 isolates were also included to aid the comparison. Six substitutions were observed in all the recent Florida clade 1 sequences when compared with the OIE-recommended Florida clade 1 strains: I8M, V35A, R260K, E271G, S337N and G416. The additional substitution N205S was present only in the sequences from Brazil and Dubai, both related to the South American outbreak of 2012. The earlier vaccine strain Kentucky/97 showed 15 changes when compared to the OIE recommended strains, and 17 changes in comparison to the isolate Rio Grande do Sul/1/12. The South American isolates from 2012 were most similar to 2011 isolates from the USA, the majority of recent American strains from 2012–2013 had three additional substitutions within NA: R252K, D258N and T434K. Taken together, these data suggest that the EIV responsible for the 2012 outbreak in South America may have originated in the USA in 2011.

**Fig. 7:**
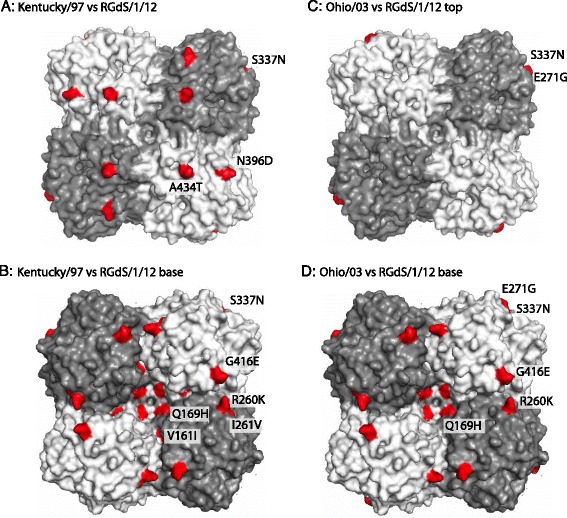
NA structure, showing locations of amino acid substitutions between A/equine/Rio Grande do Sul/1/12 NA and Kentucky/97 (**a**, **b**) and Ohio/03 (**c**, **d**). Top and bottom views are shown, with substitutions indicated in red using an N1 backbone, protein database number PDB: 2HTY [45]. The residues are numbered according to the N8 sequence. The substitutions N205S, S359A, L371F, G424V (K/97), and N205S (Ohio/03) were buried within the NA structure

The locations of individual mutations were mapped on the three dimensional structure for avian N1 NA, the closest to N8 that has been resolved as a tetramer (Fig. 7). As for HA, comparison of the outbreak strain against both the OIE-recommended vaccine strain Ohio/03 and Kentucky/97 is shown. Alignments between avian N1, avian N8 and equine N8 sequences were generated to allow determination of the correct numbering (data not shown).

### Comparison of other viral components

The derived amino acid sequences from the six segments encoding PB2, PB1, PA, PA-X, NP, M1, M2, NS1, NS2-NEP and PA-X of Rio Grande do Sul/1/12 were aligned with the corresponding amino acid sequences for the OIE-recommended strain Ohio/03 and recent isolates Kentucky/1/11 and Dubai/1/12, the observed amino acid changes in the alignments are summarised in Table 2. All sequence files were deposited with GISAID (Global Initiative on Sharing All Influenza Data), accession numbers are provided in supplementary data, Additional file 1: Table S1. Comparison of sequences of Dubai/1/12 and Rio Grande do Sul/1/12 against Ohio/03 and Kentucky/1/11 indicated that the recent Florida clade 1 strains had acquired non-synonymous mutations in seven of the eight segments compared to the OIE-recommended strain from 2003. No amino acid changes were observed in the viral nucleoprotein. The South American strains were very similar to the 2011 USA isolate, with only two additional mutations: PB2 M66V observed in Dubai/1/12 and PA-X K227R in both Dubai/1/12 and Rio Grande do Sul/1/12.

**Table 2:** Amino acid differences between recent Florida clade 1 isolates and Ohio/03

Gene	PB2									PB1					PA						PA-X			M1	M2			NS1		NEP/NS2		
aa position	63	66	212	398	660	667	684	686	731	94	200	584	621	644	59	259	348	354	409	465	59	227	240	15	59	76	87	66	209	52	64	86
Ohio/03	I	M	I	I	K	V	A	V	I	F	V	R	K	V	E	P	L	T	S	I	E	K	A	V	L	Y	E	E	N	M	K	R
Kentucky/1/11	V	.	V	V	R	I	T	I	V	L	I	Q	R	I	K	S	I	I	N	V	K	.	D	I	M	F	D	K	I	L	R	K
Dubai/1/12	V	V	V	V	R	I	T	I	V	L	I	Q	R	I	K	S	I	I	N	V	K	R	D	I	M	F	D	K	I	L	R	K
RGdS/1/12	V	.	V	V	R	I	T	I	V	L	I	Q	R	I	K	S	I	I	N	V	K	R	D	I	M	F	D	K	I	L	R	K

### Antigenic characterisation

Antigenic characterization of the Brazilian isolates was carried out by haemagglutination-inhibition (HI) assay. The reactivity of Rio Grande do Sul/1/12 (RGdS/1/12) isolate against a panel of post-infection sera raised in ferrets was compared against reference strains (Table 3). The highest titres for the Brazilian isolate were obtained against the Florida clade 1 sera, at 724–1024 these were similar to or higher than those for homologous antigens Dorset/09 and South Africa/4/03. The Florida clade 2 antisera gave titres of 128 and 256, 2 to 4- fold lower against the Brazilian isolates than to the homologous Florida clade 2 antigens. As expected, the serum against European representative Newmarket/2/93 reacted poorly against RGdS/1/12, as did the Kentucky antiserum. These showed low titres against RGdS/1/12 and the other Florida clade 1 strains that were 8- to 10- fold lower than those obtained against the homologous strains. The ferret serum raised against the Brazilian strain recognized the FC1 strains better than FC2, Kentucky or European strains. HI assays thus confirmed that the isolates from Brazil were antigenically related to viruses belonging to Florida clade 1, as predicted from their HA sequences. The viruses tested were recognised well by sera raised against the OIE-recommended strain South Africa/4/03 and the serum raised against the Brazilian strain recognised other FC1 strains, providing no evidence for significant antigenic drift.

**Table 3:** Antigenic analysis of Rio Grande do Sul/1/12 by HI assay

Ferret antisera	New/2/93	New/1/93	Rich/1/07	Dev/11	SA/4/03	Dor/09	RGdS/1/12
	Eur	Ky	FC2	FC2	FC1	FC1	FC1
Virus strain							
Newmarket/2/93	*512*	64	128	64	<8	8	8
Newmarket/1/93	16	*512*	512	128	64	32	64
Richmond/1/07	32	128	*512*	256	128	256	128
Devon/11	128	256	1024	*512*	256	256	256
South Africa/4/03	32	16	128	128	*1024*	1024	1024
Dorset/09	32	16	128	64	1024	*512*	512
Rio Grande Do Sul/1/12	32	16	256	128	1024	1024	*724*

Representative post-infection ferret sera were used against European (Eur), American (Am) and Florida clades 1 and 2 (FC1 and FC2) lineages and sub-lineages and Rio Grande do Sul/1/12

Homologous titres are shown in bold italics. New/1/93 - Newmarket/1/93, New/2/93 - Newmarket/2/93, Rich/1/07 - Richmond/1/07, Dev/11 - Devon/11, SA/4/03 - South Africa/4/03, Dor/09 - Dorset/09, RGdS/1/12 - Rio Grande do Sul/1/12

## Discussion

Large-scale epidemics of EIV have been reported since the first transmission of H3N8 in horses in the early 1960s. In some instances these have occurred in immunologically naïve populations, such as the outbreak in South Africa in 2003 and that in Australia in 2007. Epidemics that also affect vaccinated animals suggest that significant antigenic drift away from vaccine strains may have occurred. In 2012, a Florida clade 1 EIV spread through several countries in South America, affecting both vaccinated and unvaccinated animals. In some cases, animals were vaccinated with products that contained outdated strains that did not comply with current OIE recommendations, it is therefore not surprising that these animals showed clinical signs. However, animals vaccinated with a whole inactivated product containing an OIE-compliant Florida clade 1 virus were also affected. Although detailed vaccination records were not available, this is an early indication that the OIE recommendations for this sub-lineage may need updating in the near future.

Antigenic mismatches have been a major variable in well-documented vaccine breakdowns worldwide [14, 36, 37]. In horses immunized with inactivated vaccines, there is a high correlation between the level of antibodies against HA, determined by single radial haemolysis assay, and the level of protection. However, this applies in cases where the infecting EIV is homologous to the vaccine antigen [38]. When the infecting virus is antigenically heterologous, the mismatch between the vaccine and the virus can decrease the vaccine efficacy, and consequently higher levels of circulating antibodies are needed in order to neutralize the infection [39]. In such instances, vaccines may continue to offer protection against clinical signs but not necessarily reduce virus shedding and these animals become a potential source of infection to susceptible horses [40]. In the absence of serological data we cannot rule out the possibility that vaccinated animals had poor antibody titres, which may have played a part in the outbreak.

Here we describe genetic and antigenic analysis, including determination of the full genome sequence, for a representative isolate of the outbreak in Brazil in 2012. Comparison of HA and NA sequences indicated that there were multiple substitutions between the glycoproteins of these viruses and the OIE-recommended strain South Africa/4/03, however those in HA did not fulfil the much-quoted requirement for ‘four or more changes affecting two or more antigenic sites in HA1’. Nor were there multiple changes in regions previously shown to be important for EIV [37]. Substitutions of interest included one within antigenic site A of H3 (A138S) and a change within the 220 loop of the receptor-binding region (I223V), which may alter avidity for sialic acid receptors. The reciprocal substitution V223I has been observed in some recent human H3N2 clinical isolates [41, 42]. None of the amino acid changes observed here were unique to the viruses circulating in South America and were typical of those seen in USA isolates from 2011 onwards, which did not cause large scale epidemics or reports of mass vaccine breakdown.

The results of antigenic analysis using a ferret antisera panel supported the phylogenetic data, with the Brazilian isolates showing good recognition by sera raised against other members of Florida clade 1. The converse also applied, with a ferret serum raised against Rio Grande do Sul/1/12 reacting well against earlier members of the Florida clade 1 sub-lineage. Recognition by sera against Kentucky sublineage viruses was poor, similar to results for earlier FC1 viruses [10, 23]. These data do not suggest that there has been extensive antigenic drift away from the recommended OIE reference strain South Africa/4/03. Despite this, vaccines containing the recommended strain South Africa/4/03 did not perform as well as expected.

Several of the vaccines available in Brazil during 2012 contained outdated strains, including Kentucky/1/92, Kentucky/94 and Kentucky/97. The antigenic analyses described here indicated that high titres were only obtained with sera from Florida clade 1 strains, not from sera against the Kentucky sub-lineage. Vaccine breakdown with products containing these strains provide field data in support of the decision by the OIE to recommend inclusion of both Florida clade 1 and Florida clade 2 strains. More worryingly, horses that had been vaccinated with South Africa/4/03 were also infected and showed clinical signs. This may have been a consequence of antigenic mismatch with the Rio Grande do Sul/2012 strain, although the HI using ferret antisera had shown an efficient cross-reaction between both strains. However, high levels of cross-reaction are not necessarily correlated to cross-protection [36, 39]. Alternatively, poor vaccine performance may have been due to a lack of potency or poor adherence to vaccine manufacturer’s recommendations, rather than antigenic drift. Several other risk factors have been associated with influenza infection of vaccinated horses during the last decade [43]. In this instance, the use of aluminum hydroxide as an adjuvant may be relevant, as seen in the UK outbreak of 2003 ([39], Table 1). Simple adjuvants such as this may require a closer match between vaccine antigen and outbreak strain in order to be effective than more complex adjuvants that stimulate cell-mediated immunity [43].

The entry point for equine influenza into Brazil in 2012 is not known, nor has the precise origin of the South American outbreak virus been determined. Based on the reports cited here, after notification by Chile, the outbreak spread quickly across the south of the continent to Uruguay and Southern Brazil then further north. Phylogenetic analysis of HA and NA here and HA1 sequences reported recently for Argentina and Uruguay [29] showed that South American isolates from 2012 were closely related to the 2011–12 isolates from the USA. These data suggest that these viruses are likely to have originated from the USA, but through possible importation to Chile rather than gradual spread north to south.

## Conclusions

The equine influenza epidemic in South America in 2012 was caused by a virus belonging to FC1, similar to those circulating in the USA in the previous year. Phylogenetic analysis indicated that strains from FC1 had acquired several mutations in both HA and NA compared to the OIE recommended strains from 2003, however antigenic assessment using ferret antisera did not identify extensive antigenic drift. In Brazil, the use of vaccine products containing outdated strains may have contributed to the outbreak, since most antigens available on the market fell into this category. However, EIV was also isolated from clinically affected horses vaccinated with an OIE recommended strain. Poor control of horse movements following competition events is likely to have contributed to the spread of EIV.

### Consent for publication

Not applicable.

### Availability of data and materials

All novel sequence data generated have been deposited with the influenza virus sequence database, GISAID. Accession numbers are provided in Additional file 1: Table S1.

## Additional file

Additional file 1: Table S1. GISAID EpiFlu database accession numbers for EIV segments. (DOCX 43 kb)

## Abbreviations

EIV: equine influenza virus
FC1: Florida clade 1
FC2: Florida clade 2
GISAID: global initiative on sharing all influenza data
HA: haemagglutinin
HI: haemagglutinin-inhibition
NA: neuraminidase
OIE: Office International des Epizooties (World Organisation for Animal Health)
RGdS: Rio Grande do Sul
