# Supplementary material for: Characterisation of the epidemic strain of H3N8 equine influenza virus responsible for outbreaks in South America in 2012
Source: Virol J. 2016 Mar 19;13:45. doi: 10.1186/s12985-016-0503-9 (PMC4799594; doi:10.1186/s12985-016-0503-9)
Supplement: Additional file 1: Table S1. — GISAID EpiFlu database accession numbers for EIV segments. (DOCX 43 kb) [file 12985_2016_503_MOESM1_ESM.docx]

Table S1 GISAID accession numbers

| Country | Virus name | Gene segment | Accession number |
| --- | --- | --- | --- |
| Brazil | Rio Grande do Sul/1/12 | PB2 | EPI584292 |
|  |  | PB1 | EPI584293 |
|  |  | PA | EPI584294 |
|  |  | HA | EPI584295 |
|  |  | NP | EPI584296 |
|  |  | NA | EPI584297 |
|  |  | M | EPI584298 |
|  |  | NS | EPI584299 |
| USA | Kentucky/1/12 | NA | EPI670003 |
|  | New Hampshire/1/13 | NA | EPI670004 |
|  | New Hampshire/2/13 | NA | EPI670005 |
|  | Ohio/1/13 | NA | EPI670006 |
|  | Ohio/2/13 | NA | EPI670007 |
|  | Oregon/1/13 | NA | EPI670008 |
|  | Oregon/3/13 | NA | EPI670009 |
